# HERVs New Role in Cancer: From Accused Perpetrators to Cheerful Protectors

**DOI:** 10.3389/fmicb.2018.00178

**Published:** 2018-02-13

**Authors:** Norbert Bannert, Henning Hofmann, Adriana Block, Oliver Hohn

**Affiliations:** HIV and Other Retroviruses, Robert Koch Institute, Berlin, Germany

**Keywords:** human endogenous retrovirus (HERV), HERV, HERV-K, cancer, innate sensing, DNA-methylation, viral mimicry

## Abstract

Initial indications that retroviruses are connected to neoplastic transformation were seen more than a century ago. This concept has also been tested for endogenized retroviruses (ERVs) that are abundantly expressed in many transformed cells. In healthy cells, ERV expression is commonly prevented by DNA methylation and other epigenetic control mechanisms. ERVs are remnants of former exogenous forms that invaded the germ line of the host and have since been vertically transmitted. Several examples of ERV-induced genomic recombination events and dysregulation of cellular genes that contribute to tumor formation have been well documented. Moreover, evidence is accumulating that certain ERV proteins have oncogenic properties. In contrast to these implications for supporting cancer induction, a recent string of papers has described favorable outcomes of increasing human ERV (HERV) RNA and DNA abundance by treatment of cancer cells with methyltransferase inhibitors. Analogous to an infecting agent, the ERV-derived nucleic acids are sensed in the cytoplasm and activate innate immune responses that drive the tumor cell into apoptosis. This “viral mimicry” induced by epigenetic drugs might offer novel therapeutic approaches to help target cancer cells that are normally difficult to treat using standard chemotherapy. In this review, we discuss both the detrimental and the new beneficial role of HERV reactivation in terms of its implications for cancer.

## Introduction

**FIGURE 1:**
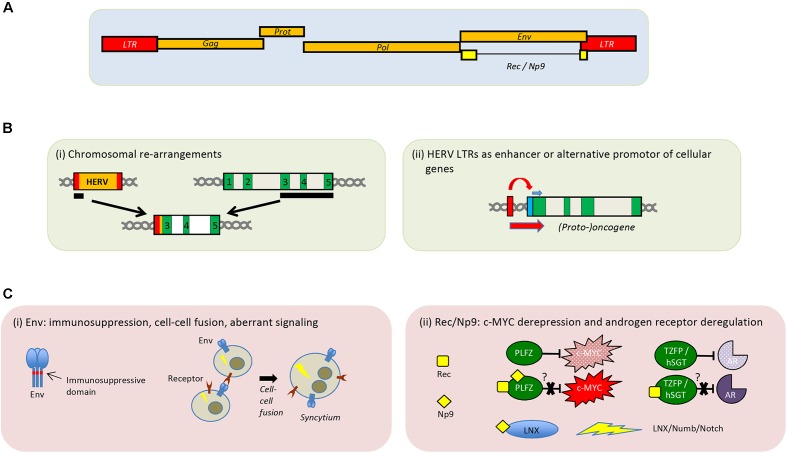
Genomic organization of HERV-K(HML-2) and proposed role of HERVs in tumorigenesis. **(A)** Schematic depiction of the HERV-K(HML-2) provirus. **(B)** Presumed cancer promoting implications of HERVs at the DNA level. (i) Recombination event leading to an LTR-driven expression of exons of a potential oncogene. (ii) LTR-driven expression of a nearby proto-oncogene. Exons are shown in green and the cellular promotor in blue. **(C)** Potential cancer-promoting effects by HERV-encoded proteins Env (i) and Rec/Np9 (ii). Aberrant signal transduction leading to transformation of the cell is indicated by a lightning sign. Binding of Rec or Np9 to PLZF prevents PLZF from functionally inhibiting c-MYC. Interaction of Rec with TZFP or hSGT has been suggested to abrogate inhibition of the androgen receptor by these two cellular proteins. Interaction of Np9 with LNX activates the LNX/Numb/Notch pathway.

Scattered throughout the genomes of all vertebrates are millions of footprints from past invasion events by retroelements; i.e., fragments of genomic DNA that have been retrotranscribed from RNA. Indeed, 43% of the human genome is made up of such elements and 8% of the genome is comprised of retroviruses that infected human ancestors, entering cells of the germ line or proliferating thereafter by retrotransposition (Katzourakis et al., 2005). This “retroviral self” can be classified into more than 30 distinct HERV families (Bannert and Kurth, 2004). By now, all of the known proviral sequences in the human germ line have suffered postinsertional mutations and deletions and have lost the ability to produce replication competent viral particles. However, around 100 of the germ line invaders belonging to the most recently active HERV-K(HML-2) family are full-length (or nearly) (**Figure 1A**). Many of the most recently acquired elements are polymorphic, leading to a diversity of haplotypes in the human population. HERV-K113, for example, a well-studied non-infectious full-length provirus with open reading frames, is present in only about 30% of Africans and 12% of individuals from other parts of the world. It is becoming increasingly evident that differences in our personal “HERVome” heritage can influence individual traits and susceptibility to disease.

This is particularly obvious with regard to the impact of retroviral promoters, splice sites and other regulatory elements on the expression of proteins and non-coding RNAs. Retroviral LTRs are *bona fide* promotors able to initiate transcription if appropriate transcription factors are present in the nucleus and their access to the LTR is not epigenetically restricted. Under such conditions, mRNAs are produced that occasionally encode functional viral proteins, and in the case of HERV-K(HML-2), non-infectious viral particles are in fact released (Boller et al., 1983). In differentiated healthy cells, however, LTR activity is tightly repressed by epigenetic constraints such as DNA methylation. In contrast, silencing in embryonic stem cells depends primarily on the activity of histone methyltransferases and other histone modifications (Rowe and Trono, 2011). Transcription of retroviral LTRs plays a fundamental role in the maintenance of pluripotency and induction of an antiviral state in those cells (Grow et al., 2015). The physiological role of HERV expression in embryonic stem cells is not the only known example of domestication of these genomic parasites to serve the host, i.e., “exaptation.” The best known examples in this regard are the *syncytin* genes: HERV envelope proteins under positive selection that play an important role in the physiology of the placenta in mammals (Dupressoir et al., 2012; Lavialle et al., 2013).

Conversely, since the early days of HERV research, these elements have been implicated in cellular transformation processes associated to various types of cancer, although recent studies suggest that expression of HERV-derived nucleic acids may also have a beneficial impact in the fight against cancer.

### Implications of HERVs in the Promotion of Transformation

Investigation on human retroviruses and their involvement in cancerogenesis started in the early 1970s with the search for reverse transcriptase activity and virus particles in tumor cells (Sarngadharan et al., 1972; Zhdanov et al., 1973). This search was later extended to retroviral sequences derived from or related to murine retroviruses in the human genome, as several murine retroviruses are established transforming agents (Chumakov et al., 1982; Repaske et al., 1983).

There is a plethora of publications reporting HERV activation in various cancers: breast cancer (Wang-Johanning et al., 2001, 2003, 2008; Burmeister et al., 2004; Contreras-Galindo et al., 2008; Golan et al., 2008; Zhou et al., 2016; Johanning et al., 2017), lymphoma (Contreras-Galindo et al., 2008; Maliniemi et al., 2013; Fava et al., 2016), melanoma (Muster et al., 2003; Buscher et al., 2005; Hirschl et al., 2007; Serafino et al., 2009; Reiche et al., 2010; Stengel et al., 2010; Huang et al., 2013; Singh et al., 2013), ovarian cancers (Gotzinger et al., 1996; Wang-Johanning et al., 2007; Iramaneerat et al., 2011; Heidmann et al., 2017), and prostate cancers (Tomlins et al., 2007; Ishida et al., 2008; Goering et al., 2011; Agoni et al., 2013; Goering et al., 2015). However, to date, there is no conclusive picture emerging regarding the role and impact of HERVs as causative or promoting agents in cancerogenesis, although some well-described examples of links at the DNA and protein levels are known.

#### At the DNA Level

Non-allelic recombination of HERV sequences can lead to deletions, duplications, and other chromosomal rearrangements (**Figure 1B**). In some prostate cancer cases, a translocation of the HERV-K_22q11.23 5′-LTR-UTR sequence upstream of the transcription factor ETS translocation variant 1 (ETV1) has been described, which results in the enhanced expression of the ETV1 oncogene promoting cancerogenesis (Tomlins et al., 2007). LTRs can also act as alternative promotors and dysregulate nearby proto-oncogenes, or growth-promoting cellular genes (**Figure 1B**). For example, it was shown in B cell-derived Hodgkin’s lymphoma cells that transcription of the proto-oncogene colony-stimulating factor 1 receptor (CSF1R) is driven by an aberrantly activated LTR promoter of the THE1B retrotransposon, an apparent member of the mammalian LTR retrotransposons (MaLR) family (Lamprecht et al., 2010). Moreover, in the same study, it could also be demonstrated that the derepression of THE1B LTR in those Hodgkin’s lymphoma cells is a consequence of the loss of transcriptional corepressor CBFA2T3 expression, which leads to disturbed epigenetic control (Lamprecht et al., 2010). A very recent review summarizes several studies addressing the impact of HERV LTR on cellular genes, among other effects (Kassiotis and Stoye, 2017).

#### At the Protein Level

Unlike HTLV-1 and the *tax* protein, HERVs do not possess a *bona fide* oncogene. However, expression of some HERV Env proteins may also be detrimental due to its ability to induce cell–cell fusion and may contribute by this way or others to tumorigenesis (Duelli and Lazebnik, 2003; Bjerregaard et al., 2006). In *in vitro* experiments, an Env protein coded by the HERV-K(HML-2) consensus sequences as well as by several HERV-K(HML-2) elements can interact with a cellular signaling pathway often involved in cancers (Lemaitre et al., 2017), suggesting a potential pro-oncogenic role of these HERV Envs (**Figure 1C**). Another potential effect of HERV Env expression might be the promotion of tumor escape through immune modulation, mediated by the immunosuppressive domain contained in the transmembrane region (Mangeney et al., 2001, 2005; Kudo-Saito et al., 2014).

Moreover, the expression of the HERV-K(HML-2) accessory proteins Rec and Np9 (Magin et al., 1999; Armbruester et al., 2002) can be linked to tumorigenesis (Galli et al., 2005; Chen et al., 2013; Schmitt et al., 2013; Singh et al., 2013; Fischer et al., 2014). Paradoxically, transcripts of *rec* and *np9* from different HERV-K(HML-2) loci appear to be present in various normal human tissues (Schmitt et al., 2015). It is known that Rec and Np9 both interact with the cellular promyelocytic leukemia zinc-finger protein (PLZF) (Boese et al., 2000; Denne et al., 2007), a transcriptional repressor of the c-MYC proto-oncogene. Furthermore, Rec also binds to the testicular zinc-finger protein (TZFP) (Kaufmann et al., 2010) and the human small glutamine-rich tetratricopeptide repeat protein (hSGT) (Hanke et al., 2013), both involved in androgen receptor repression (**Figure 1C**). Rec-driven dysregulation of the androgen receptor signaling may eventually result in tumor induction or promotion (Hanke et al., 2013). In addition to PLZF, Np9 also binds the ligand of Numb protein X (LNX) and is therefore interacting with the Numb/Notch signaling cascade (Armbruester et al., 2004). Dysregulation of this pathway has been linked to several cancers (Roy et al., 2007; Downey et al., 2015).

### HERV Expression as a Diagnostic Marker for Tumors

The significantly elevated expression of various HERV elements in cancer cells has spurred studies for their use as biomarkers for malignant transformation, staging, and prognosis of cancers (Harzmann et al., 1982; Hahn et al., 2008; Wallace et al., 2014; Perot et al., 2015; Ma et al., 2016). One of the best candidates for diagnostic purpose in this regard is the HERV-K(HML-2) envelope protein in human breast cancer. Zhao et al. (2011) demonstrated that this gene is expressed in the majority of breast cancers from United States or Chinese women but generally not expressed or at very low levels in normal breast tissue. They subsequently showed that HERV-K(HML-2) antibodies and mRNA are elevated in blood of patients at an early stage of this cancer type, and further increase in patients who are at risk of developing metastatis (Wang-Johanning et al., 2014). Thus, screening for HERV-K(HML-2) expression seems to be a promising additional option for early detection in women at increased risk for breast cancer.

### Antitumor Activity of HERVs

In many respects, an endogenous retrovirus is an intermediate between a genuine virus and a regular human gene. This also applies to the immune response directed against HERV-derived nucleic acids and proteins.

#### Adaptive Immunity

Immunologic tolerance to HERV-derived proteins and peptides is imperfect. This is presumably due to the tight epigenetic silencing in the thymus and bone marrow that prevents normal deletion of all reactive HERV-specific T and B lymphocytes, respectively. Indeed, immunization of non-human primates with endogenous retrovirus-derived antigens elicits robust polyfunctional T cell responses and high antibody titers (Sacha et al., 2012; Sheppard et al., 2014). In line with these findings, Boller et al. (1997) were among the first to report transient HERV-K(HML-2)-specific antibodies in the plasma of testicular cancer patients that became rapidly undetectable following tumor surgery. Strong CTL responses against epitopes of certain HERV proteins, considered to be another class of tumor-specific antigens, have been found in patients with various types of cancers (Schiavetti et al., 2002; Mullins and Linnebacher, 2012; Rycaj et al., 2015). Although evidence for tumor regression by the action of HERV-specific CTLs exists, the general impact of these antigens on the adaptive antitumoral defense remains largely unclear (Huang et al., 1996; Takahashi et al., 2008). This also holds true for the potential use of tumor-specific HERV-based therapeutic vaccines against various types of cancers.

#### Innate Immunity

**FIGURE 2:**
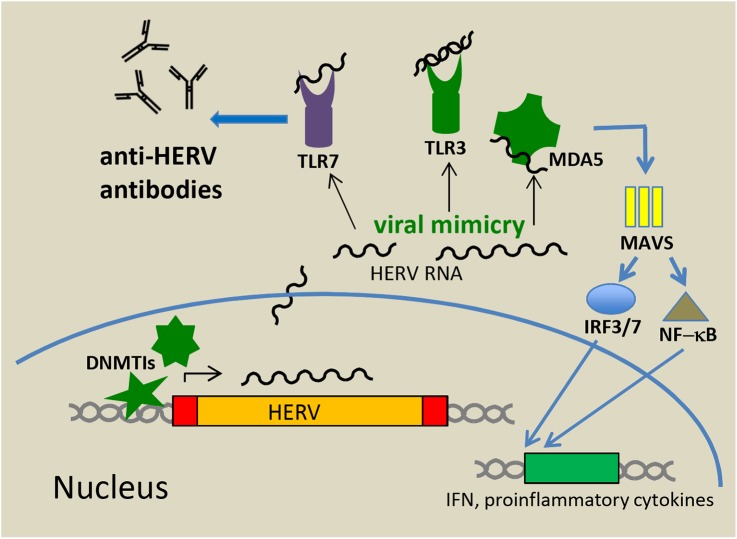
Antitumor activity mediated by innate sensing of HERV RNA and DNA. Treatment of cancer cells with DNMTis results in demethylation of the LTR promotors and HERV transcription. Viral nucleic acids in the cytoplasm and other cellular compartments (viral mimicry) are sensed by TLRs and other innate sensors including MDA5. Upon activation these pattern recognition receptors initiate signal transduction pathways that result in production of type I and III IFNs and pro-inflammatory cytokines further leading to promotion of an adaptive immune response (e.g., anti-HERV antibodies).

The nucleic acids derived from endogenous retroviruses in the cytoplasm and other cellular compartments do not escape a response from the innate immune system. Although there are marked differences between the innate immunity of humans and mice, similar principles might act during the control of ERV reactivation and innate sensing. In an elegant study in a mouse model, Yu et al. (2012) have shown that Toll-Like Receptors (TLRs) 3, 7, and 9 are essential for the control of ERVs at least in mice. Mice lacking these receptors develop late-onset leukemia by insertional mutagenesis of reactivated replicating ERVs (Yu et al., 2012). A key factor for the production of anti-ERV antibodies was further attributed to TLR7 thereby linking innate and adaptive immunity (**Figure 2**). Similar to TLR-knockout mice, those with inactivating mutations in the maintenance gene DNA methyltransferase 1 (DNMT1) develop tumors induced by reactivation of replication-competent ERVs (Howard et al., 2008). Significant DNA hypomethylation and ERV activation can also be achieved by treatment with DNA methyltransferase inhibitors (DNMTis), such as 5-azacytidine (Aza) and 5-aza-2′-deoxycytidine (Dac) (Stengel et al., 2010), both of which are approved by the FDA for the treatment of myelodysplastic syndromes (Kaminskas et al., 2005). These inhibitors were initially thought to epigenetically reactivate silenced tumor suppressor genes in malignant cells and render these cells therefore more prone to apoptosis, but it is also recognized that DNMTis induce immune responses against cancer cells. Chiappinelli et al. (2015) and Roulois et al. (2015) demonstrated a link between DNMTi-induced activation of HERV expression and innate sensing of transcribed viral RNAs and activation of innate immunity signaling pathways leading to an inhibition of tumor cell growth. These results represent a paradigm shift in our comprehension of the antitumor activity of demethylating agents. The authors demonstrated that DNMTis induce HERV-derived dsRNAs that are sensed primarily by TLR3 (localized at the endosomal membrane) and by the cytosolic CARD-domain family protein MDA5, a known pattern recognition receptor. Following dsRNA binding, the CARD domain of MDA5 interacts with the mitochondrial antiviral signaling protein MAVS located on mitochondrial membranes. This leads to the activation of NF-kB and the phosphorylation and nuclear import of IRF7 resulting in a profound interferon (IFN) response (**Figure 2**). TLR3 binding to dsRNAs of endogenous origin also leads to both IRF and NF-kB activation. In these experiments, Aza treatment also induced partial demethylation of the IRF7 gene and increased its expression. Due to the slow response toward DNMTi treatment, the type I IFN response and thereby IFN release and activation of numerous IFN Stimulated Genes (ISGs) are delayed and peak about 1 week after treatment (Chiappinelli et al., 2015). Interestingly, while in the ovarian cancer cell model, the outcome of a low dose Aza treatment was dominated by an IFN I response (Chiappinelli et al., 2015; Stone et al., 2017), in colorectal cancer cells, activated IFN III genes were more preponderant (Roulois et al., 2015). The latter group also demonstrated that cancer initiating cells, defined by their ability to self-renew and difficult to reach by standard chemotherapy, were importantly targeted by treatment. The overall outcome of the innate immune response was a suppression of tumor cell proliferation and enhanced apoptosis. Moreover, Aza administration sensitized murine melanoma cells to anti-CTLA-4 treatment and high expression of ISGs correlated with a sustained clinical response to anti-CTLA-4. A treatment combining DNMTis and immune checkpoint inhibitors (e.g., anti-CTLA-4) is therefore regarded as extremely promising. Supplementation with vitamin C was also suggested to increase the effect of the DNMTis and was shown to be effective (Liu et al., 2016). Vitamin C promotes DNA demethylation through increased activity of the three so-called “Ten-Eleven Translocation (TET)” enzymes, which convert 5-methylcytosine to 5-hydroxymethylcytosine (Tahiliani et al., 2009).

The induction of immune responses by unleashing ERV expression from epigenetic restrictions has been termed “viral mimicry,” i.e., a cellular response similar to those seen after infection with an exogenous virus (**Figure 2**). Results from these studies using Aza treatment have been corroborated by others in different settings (Nicholas et al., 2017). In one of these studies, a 3D culture system of intestinal mice tumors was used to demonstrate that DNMT knockdown by Aza treatment significantly reduced cell proliferation in tumor organoids (Saito et al., 2016). More recently, the anticancer agent, RRx-001, a dinitroazetidine derivate that is currently tested in phase II clinical trials, has also been shown to elicit an IFN response through epigenetic induction of viral mimicry. The remarkable safety profile of this immunomodulatory anticancer agent makes it a leading candidate for future clinical applications (Zhao et al., 2017).

Although reactivated endogenous elements have been roughly categorized at the family level, the actual chromosomal loci remained unknown. The identification of these loci might help to better understand epigenetic changes and cancer-specific differences. In this context, it might also explain the surprisingly frequent occurrence of bidirectional transcription in many ERVs which appears to be the underlying reason for the strong activation of MDA5, as this innate sensor requires extended dsRNA structures for efficient activation (Mu et al., 2016).

There are many additional options for the enhancement and advancement of an ERV-mediated epigenetic cancer therapy. One approach might be the inhibition of dsRNA or DNA degradation in cancer cells by blocking the activity of the respective nucleases. Such an accumulation of ERV nucleic acids has been reported in cells from individuals bearing inactivating mutations in nucleases that normally clear nucleic acid from the cytoplasm or influence their metabolism in the cytoplasm. The most prominent examples are mutations in Three-prime Repair Exonuclease 1 (TREX1) or Sam domain and HD domain 1 (SAMHD1), that are associated with the rare autoinflammatory disease Aicardi-Goutieres syndrome (AGS) characterized by an exaggerated type I IFN response (van Montfoort et al., 2014).

## Conclusion

It has been recognized for many years that endogenous retroviruses and other retroelements contribute to malignant diseases as well as to inflammatory and autoimmune disorders at the DNA and presumably at the protein level. However, until recently, it escaped attention that an increased expression of HERV-derived nucleic acids also has an adverse effect on cancer cells and that this effect could be the basis of novel therapeutic approaches. Importantly, targeting of the neoplastic cell will be an important issue to prevent jumping from the “frying-pan” into the fire. Aberrant reactivation and expression of HERVs in healthy tissue not only bears the risk of new transformations and autoimmune diseases, but also might influence cellular physiology by activating HERV promotors that act on cellular genes. Curing cancers by activating HERVs that instigate an innate immune response is surely an appealing concept with high expectations and is worth investing significant effort in the future.

## Author Contributions

All authors listed have made a substantial, direct and intellectual contributions to this mini review, and approved it for publication.

## Conflict of Interest Statement

The authors declare that the research was conducted in the absence of any commercial or financial relationships that could be construed as a potential conflict of interest.
